# Alaska—Klondike

**Published:** 1897-09

**Authors:** 


### ALASKA—KLONDIKE.

THE suggestion made by one of the resident missionaries of Alaska that our government would do well to stock that country with reindeer should not go unheeded. Perhaps our Bureau of Animal Industry would find this another avenue of great usefulness to our people. The animals would live, and, under proper care and direction, do well, and be a source of supply of milk, meat, and clothing, tending largely to mitigate the hardships of that country. There is sufficient for them to feed upon, and their disposition to forage under the snow fits them peculiarly for that region and its people.

We are not aware of any special information as to the susceptibility of these animals to tuberculosis, but no doubt if affected they would respond to the tuberculin-test, and the country could be stocked with animals free from this disease, and occasional examinations would keep them practically exempt. The climate of Alaska is not favorable to the multiplication of the tubercle bacilli, which adds to the worth of the suggested plan. We should not forget the parental duty of our government in conserving the best interests and welfare of the whole people, and we especially commend this suggested aid to the consideration of Secretary Wilson and Chief Salmon.

